# Neighborhood Conditions in a New Destination Context and Latine Youth’s Ethnic–Racial Identity: What’s Gender Got to Do with It?

**DOI:** 10.3390/bs15091148

**Published:** 2025-08-23

**Authors:** Olivia C. Goldstein, Dawn P. Witherspoon, Mayra Y. Bámaca

**Affiliations:** 1 Department of Psychology, The Pennsylvania State University, University Park, PA 16802, USA; dpw14@psu.edu; 2 Department of Women’s, Sexuality, and Gender Studies, The Pennsylvania State University, University Park, PA 16802, USA; 3 Department of Psychological Sciences, University of California, Merced, CA 95343, USA; mbamaca@ucmerced.edu

**Keywords:** ethnic–racial identity, gender role socialization, parental socialization, neighborhood context, Latine or Latino families, adolescent development, cultural parenting beliefs, new destination communities

## Abstract

This exploratory pilot study examined how Latine adolescents’ ethnic–racial identity (ERI)—specifically, centrality, private regard, and public regard—was shaped by parents’ gender role socialization (GRS) beliefs and perceptions of neighborhood connectedness and problems. Sixty Latine parent–adolescent dyads living in a Northeastern new destination context participated. Hierarchical regression models were used to test whether GRS beliefs moderated the effects of neighborhood on adolescents’ ERI. Traditional GRS beliefs moderated associations between neighborhood problems and ERI dimensions, such that adolescents whose parents endorsed stronger traditional GRS beliefs reported lower ERI centrality, private regard, and public regard in neighborhoods with more problems. These associations were not significant for neighborhood connectedness and did not differ by child gender. Findings suggest that parent beliefs about gender may shape identity development in environments perceived as risky or under-resourced. The context-dependent nature of socialization and the adaptive nature of parenting processes in emerging Latine communities are discussed.

## 1. Introduction

Adolescence is a key period for identity formation, particularly ethnic–racial identity (ERI), which is linked to social–emotional wellbeing, academic engagement, and health outcomes among youth of color in the U.S. (Rivas-Drake et al., 2014). For Latine^1^ youth, ERI develops at the intersection of multiple socializing forces—most notably family, cultural context, and place (Witherspoon et al., 2023). As demographic shifts lead to the growth of Latine populations in new destination contexts, questions emerge about how environmental factors and parenting beliefs and practices shape ERI in places with fewer culturally specific supports.

Place is more than geography; it is a developmental context that reflects racialized histories, cultural infrastructures, and collective memory. *New destination contexts* refer to places in the U.S. that historically had low immigrant populations but experienced rapid growth in immigrant residents—particularly Latine populations—beginning in the 1990s and 2000s (Massey, 2008). Compared to traditional Latine enclaves, which are supported by robust ethnic networks, cultural institutions, and community supports, Latine families in new destination contexts must navigate both structural constraints and emerging opportunities to sustain cultural practices and transmit values (Massey, 2008; Stein et al., 2016; Marrow, 2020). Moreover, cultural-contextual frameworks of development suggest that parenting is shaped not only by individual beliefs but also by broader ecological systems—including race, ethnicity, gender, and immigrant status (Witherspoon et al., 2023; García Coll et al., 1996). How parents interpret their local context—as connected or problematic—likely shapes the messages and practices they prioritize in raising their children.

Guided by frameworks that emphasize parenting-by-context interactions (García Coll et al., 1996; White et al., 2018), this exploratory pilot study examines how Latine parents’ socialization practices—including both GRS beliefs and ERS practices—and neighborhood perceptions interact to shape adolescents’ ERI in a new destination city. Specifically, we ask the following: how do beliefs about gender roles—often shaped by heritage culture and patriarchal norms—and perceptions of neighborhood connectedness and risk work together to shape adolescents’ ERI content (centrality, private regard, and public regard)? The study focuses particularly on the former, GRS beliefs, and their effects in neighborhoods, specifically with more perceived problems.

### 1.1. Theoretical Frameworks

The current study draws on three interrelated theoretical frameworks to examine how socialization (i.e., GRS beliefs and ERS practices) and context (i.e., neighborhood) shape Latine adolescents’ ERI: the Integrative Model of child development, pluralistic neighborhood theory, and the Adapting Cultural Systems framework.

The Integrative Model (García Coll et al., 1996) emphasizes that development for youth of color occurs within systems structured by social position factors such as race, ethnicity, immigrant status, and gender. These structural hierarchies create segregated environments—residential, economic, social, and psychological—that shape both opportunities and constraints. Within these stratified contexts, parents adapt their socialization strategies to support their children’s development. For example, parents’ perceptions of their neighborhood—as either supportive or threatening—may guide what values or warnings they prioritize when socializing their children (Witherspoon et al., 2023).

Pluralistic neighborhood theory (Aber & Nieto, 2000) complements this model by recognizing that neighborhood environments are complex and multidimensional. Structural risks (e.g., crime, poverty, dilapidation) and social resources (e.g., cohesion, trust, mutual support) can coexist in the same community. Even in neighborhoods marked by disinvestment, positive relational processes can serve as promotive forces for youth development. Importantly, perceptions of neighborhood quality are subjective and may differ by generation, gender, or immigrant status. This study considers both positive (social connectedness and cohesion) and negative (perceived problems and disorder) aspects of neighborhoods as reported by parents.

To integrate cultural heritage with environmental context, we also draw on the Adapting Cultural Systems framework (White et al., 2018). This framework bridges ecological (e.g., Benner & Kim, 2010) and cultural (e.g., Bornstein, 2013) perspectives, proposing that parenting practices and developmental outcomes are shaped by both historical legacies and current structural realities. It foregrounds the role of heritage culture and environmental adaptation in shaping family processes, offering a nuanced lens for interpreting group-level variation in parenting across neighborhoods. This framework is particularly useful for understanding parenting-by-context interactions in minoritized families, such as how traditional gender beliefs may interact with neighborhood perceptions to shape ERI outcomes in youth. Together, these frameworks situate ERI development as a dynamic process shaped by intersecting systems—social positions, environment, and culture—and guide our examination of how Latine parents’ GRS beliefs and neighborhood perceptions influence adolescents’ ERI context in a new destination context.

### 1.2. Ethnic Racial Identity and Socialization in Context

Ethnic–racial identity (ERI) is shaped not only by developmental *processes* like exploration and resolution but also by the *content* of identity—how adolescents evaluate and center their racial-ethnic group membership (Umaña-Taylor et al., 2014; Phinney et al., 2001). For Latine adolescents in the U.S., ERI content is linked to cultural affirmation, belonging, and good health in the face of racial marginalization (Huguley et al., 2019). The current study focuses on three aspects of content, including *centrality*, the extent to which one considers their ethnic–racial identity to be an important aspect of their self-concept; *private regard,* the positive affect one feels towards their ethnic–racial group; and *public regard*, the affect one perceives others feel towards their ethnic–racial group (Umaña-Taylor et al., 2014; Sellers et al., 1997).

Among Latine families, ethnic–racial socialization (ERS) practices—used to communicate messages about race, culture, and discrimination (Hughes et al., 2006)—have been consistently linked to ERI development, particularly centrality and private regard (Huguley et al., 2019; Umaña-Taylor & Hill, 2020; Ayón et al., 2020; Else-Quest & Morse, 2015; Sanchez et al., 2017). Past findings suggest that these practices—and their impact—may vary depending on youth gender and parental beliefs (Miville et al., 2017; K. M. Schroeder & Bámaca-Colbert, 2019; Raffaelli & Ontai, 2004). While ERS is widely recognized as a key influence on ERI development among Latine youth (Huguley et al., 2019; Umaña-Taylor & Hill, 2020; Ayón et al., 2020; Else-Quest & Morse, 2015; Sanchez et al., 2017), the current exploratory analyses focused on parents’ GRS beliefs and neighborhood perceptions as predictors of adolescents’ ERI content. Exploratory models testing ERS as a mediator are presented in Appendix A to situate the study within its broader theoretical framework and maintain transparency.

### 1.3. Gender Role Beliefs and Cultural Scripts

Gender role socialization (GRS) beliefs are theorized to shape both parenting practices (e.g., socialization) and adolescent identity outcomes (K. M. Schroeder & Bámaca-Colbert, 2019; Sanchez et al., 2016), yet they have not been studied as rigorously as ERS (e.g., Umaña-Taylor et al., 2014). GRS beliefs reflect culturally shaped expectations for behaviors, traits, and attitudes associated with masculinity and femininity (American Psychology Association, 2023; Ruble et al., 2007). In Latine communities, these beliefs are often transmitted through cultural scripts such as *marianismo* and *machismo* (Castillo et al., 2010; Rivera et al., 2024), which can inform how parents monitor youth, grant autonomy, or respond to perceived environmental threats (White et al., 2009; Piña-Watson et al., 2014). Rooted in Roman Catholic traditions and the veneration of La Virgen María (Stevens, 1973), *marianismo* promotes ideals of moral purity, self-sacrifice, nurturance, and relational subordination among Latine girls and women (Castillo et al., 2010; Rivera et al., 2024). In contrast, *machismo* encompasses a broader set of expectations for Latine men—ranging from hypermasculinity and dominance to *caballerismo*, which emphasizes chivalry, respect, and family responsibility (Rivera et al., 2024; Arciniega et al., 2008).

Notably, gendered scripts are neither inherently positive nor negative, but instead are dynamic, context-dependent, and shaped by broader sociohistorical forces (García Coll et al., 1996). For example, *marianismo* may foster community orientation and family cohesion while simultaneously encouraging self-silencing and dependence (Castillo et al., 2010; Rivera et al., 2024). Despite their complexity and cultural salience, these beliefs remain underexplored in empirical studies of Latine family dynamics and youth identity development. Indeed, a content analysis of empirical studies from 1982 to 2013 found that while machismo and marianismo were frequently referenced, few studies deeply examined how gender role beliefs operate within families to shape adolescent development (Miville et al., 2017). It is important to understand how parents’ endorsement of traditional gender role beliefs interacts with environmental contexts to shape youth identity development in culturally grounded ways. Prior research has examined how GRS beliefs differ by individual (e.g., immigrant status, parent gender, child gender, age) and environmental (e.g., neighborhood) factors.

### 1.4. Individual-Level Influences on GRS Beliefs and ERI

Parental GRS beliefs—and accompanying ERS practices—vary considerably by individual-level factors, including parent and child gender. Across studies, Latina daughters more frequently receive traditional GRS messages than sons, particularly around appearance, behaviors, and autonomy (K. M. Schroeder & Bámaca-Colbert, 2019; Araujo Dawson & Quiros, 2014). Daughters often report awareness of double standards, such as stricter curfews and modesty expectations compared to male siblings (Gallegos-Castillo, 2006; Hurtado, 2003). In one retrospective study, Latina women described puberty as a turning point that triggered increased restrictions on dating and social freedom outside the home (Raffaelli & Ontai, 2004), reflecting how individual age and developmental period may also shape beliefs and enforcement of GRS.

These patterns are not monolithic within Latine youth. Some studies with Mexican-heritage girls report that domestic expectations begin early in adolescence, sometimes coinciding with the onset of menstruation, and tend to intensify as girls age into later adolescence (Raffaelli & Ontai, 2004). In contrast, university samples of Mexican-heritage adolescents received fewer traditional messages, especially from familial and nonfamilial peers, compared to older relatives (Gutierrez & Leaper, 2025). Similarly, a study with Colombian, Guatemalan, Mexican, and Puerto Rican mothers revealed that while familial ethnic socialization practices were relatively consistent, community resources aiding mothers’ ability to promote ethnic–racial socialization varied significantly by mothers’ national origin group, further highlighting intragroup variability in how ethnicity is taught and experienced (Umaña-Taylor & Bámaca, 2004).

Family structure and generational status further shape GRS. Traditional messages are often transmitted through older relatives (e.g., grandparents) or first-generation immigrant parents, even as youth adopt more egalitarian norms (K. M. Schroeder & Bámaca-Colbert, 2019; Hurtado, 2003; Umaña-Taylor & Bámaca, 2004). For immigrant families, maintaining cultural heritage may involve reinforcing gendered scripts at home, which can contrast with adolescents’ expectations—especially in new-destination contexts, where neighborhood norms may differ—potentially leading to tension or identity ambiguity (Manago et al., 2015; Phinney et al., 2001; Suárez-Orozco & Suárez-Orozco, 2001; Umaña-Taylor et al., 2006).

Though dominant discourse often frames U.S. culture as more gender egalitarian than Latin American cultures, such comparisons oversimplify the sociopolitical histories and changes occurring across—and between—both contexts (Williams, 2020; Vasquez, 2014). Several Latin American countries, including Chile, Argentina, and Mexico, have made notable advancements towards gender equity, including legalizing same-sex marriage, expanding access to reproductive health services, and electing women to the presidency (e.g., Michelle Bachelet in Chile, Cristina Fernández de Kirchner in Argentina, Claudia Sheinbaum Pardo in Mexico). These developments, coupled with political shifts in the United States, such as the 2025 presidential inauguration of Donald Trump, challenge dominant narratives portraying Latin American cultures as inherently more traditional or patriarchal. Still, traditional GRS beliefs, particularly among older and immigrant family members, may shape the association between individual and environmental factors and Latine adolescents’ identity development.

### 1.5. Neighborhood-Level Influences on GRS Beliefs and ERI

While much of the existing literature emphasizes the role of neighborhood environments in shaping ERS practices, comparatively less attention has been paid to how parenting beliefs—particularly GRS beliefs—inform how parents perceive their environments that may directly or indirectly shape adolescents’ ERI. GRS beliefs, reflecting culturally grounded expectations for autonomy, behavior, and responsibility based on gender, may guide parents’ judgments about what aspects of neighborhood are threatening or supportive, and what values or restrictions they emphasize in response. In this way, GRS beliefs may serve not as products of context, but as moderators of the relation between neighborhood environment and ERI content.

Research supports the idea that neighborhood contexts provide both cultural stressors (e.g., discrimination, crime) and assets (e.g., cohesion, trust) (Aber & Nieto, 2000; Witherspoon et al., 2023) but their meaning is filtered through social position and belief systems (García Coll et al., 1996; Witherspoon et al., 2023; Umaña-Taylor & Hill, 2020). For instance, Witherspoon et al. (2021) found that parents living in neighborhoods with higher levels of perceived disorder reported more frequent use of cultural socialization and prep for bias messaging than those living in more affirming neighborhoods. In contrast, parents in high affirmation contexts (i.e., Latine ethnic enclaves) emphasized cultural pride but conveyed fewer warnings about discrimination. Latine parents reported less frequent use of ERS messages compared to African American parents. Moreover, foreign-born Latine parents communicated more cultural socialization than U.S.-born counterparts and perceived more neighborhood problems—suggesting variation in how cultural values are enacted across contexts and nativity.

While Witherspoon et al. (2021) focused on ERS practices, other research points to gendered patterns that may reflect underlying GRS beliefs. For instance, Morton et al. (2024) found that preparation for bias messaging was linked to higher ERI exploration and belonging among Latino and Black early adolescents but not for boys in higher-SES neighborhoods, perhaps due to increased access to racially integrated settings. Boys in this study reported more engagement with cross-ethnic peer groups than girls, who may experience more parental monitoring and restricted peer relationships. These differences indicate that parental messages are not only shaped by structural conditions but also by gendered expectations about navigating public spaces and identity, underscoring the importance of GRS beliefs in shaping developmental outcomes across contexts.

**Figure A1:**
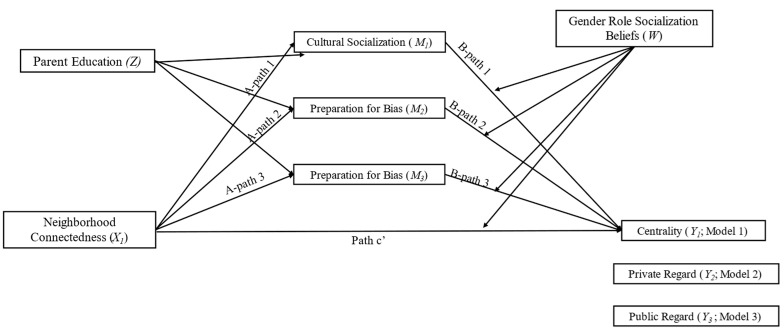
Conceptual model of neighborhood connectedness predicting ERI (i.e., *centrality*—Model 1; *private regard*—Model 2; *public regard*—Model 3) through ERS, moderated by GRS.

Together, these studies point to the need to move beyond identifying environmental risks and ERS strategies alone and, instead, examine how broader parental socialization—including GRS beliefs—shapes how parents perceive and respond to their neighborhood contexts. In particular, GRS beliefs may moderate these perceptions and responses in ways that affect adolescents’ ERI development (see Figure A1 in Appendix A). Prior research has shown that Latine parents often emphasize traditional gendered scripts, such as *marianismo* and *machismo*, especially in contexts where safety or cultural preservation is a concern (Castillo et al., 2010; K. M. Schroeder & Bámaca-Colbert, 2019). In these contexts, beliefs about daughters’ vulnerability or sons’ independence may guide how cultural pride or warnings about discrimination are communicated—thereby shaping youths’ internalization of ERI. While ERS is a well-established driver of ERI development, it is also important to consider how gendered beliefs filter parental socialization messages and structure the developmental meaning of place. The current study builds on this literature by examining whether GRS beliefs moderate the relation between parents’ neighborhood perceptions of both positive social processes and perceived problems, and adolescents’ ERI content, providing insight into how gendered belief systems filter the developmental impact of place.

### 1.6. Study Aims and Hypotheses

**Figure 1:**
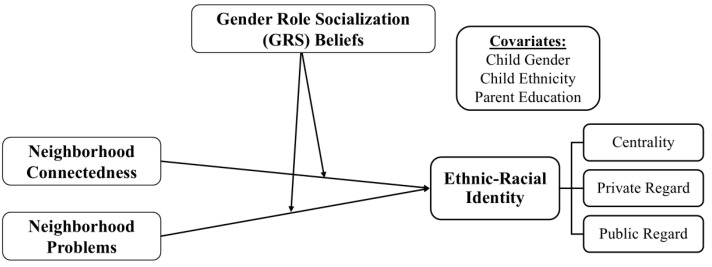
Conceptual model predicting ERI content from neighborhood variables, potentially moderated by GRS beliefs.

Prior research has not fully examined how neighborhood contexts impact adolescents’ ERI development relative to individual and environmental characteristics such as youth gender, GRS beliefs, and parents’ perceptions of their local environments. This study aims to address this gap by examining how gendered parenting beliefs (i.e., GRS beliefs) and perceptions of neighborhood context interact to shape adolescents’ ERI in a new destination context (see Figure 1 for the conceptual model). Guided by theory and past empirical research, the following hypotheses were tested.

**Hypothesis** **1.**  *Parents’ perceptions of neighborhood connectedness will be positively associated with ERI content—specifically ERI centrality and private regard. In contrast, perceptions of neighborhood problems will be negatively associated with ERI content.*

**Hypothesis** **2.**  *Adolescents whose parents hold more traditional GRS beliefs and higher perceptions of neighborhood problems will report lower ERI outcomes. In contrast, adolescents whose parents hold more egalitarian beliefs will report higher ERI outcomes, even in the presence of neighborhood problems.*

**Hypothesis** **3.**  *Girls will be more strongly affected by parents’ traditional GRS beliefs and perceptions of neighborhood problems than boys, such that these factors will be more negatively associated with self-reported ERI outcomes among girls.*

## 2. Materials and Methods

### 2.1. Participants and Procedure

The current study used data from the *Places/Lugares* pilot study, which were collected between April and December 2016 from Latine parents and adolescents living in a new destination city in central Pennsylvania. All subjects gave their informed consent for inclusion before they participated in the study. The study was conducted in accordance with the Declaration of Helsinki, and the protocol was approved by the Internal Review Board at The Pennsylvania State University (# STUDY00004644) on 29 March 2016.

The *Places/Lugares* study partnered with a Latine-serving community organization and nine other community organizations (e.g., religious, social service, educational) through the Parents And Children Together (PACT) research initiative. Families were recruited via flyers at frequented locations (e.g., laundromats, grocery stores, bodegas, restaurants) and at approximately 16 community events attended by researchers as part of the PACT partnership. Bilingual research staff (English/Spanish) conducted participant screening, obtained parent consent and adolescent assent, and proctored the questionnaires in participants’ language of choice. Most parents (81%) completed the measures in Spanish, whereas most adolescents (81%) completed them in English. Families received monetary incentives and were provided lunch and free childcare during data collection.

A total of 70 parents and 82 adolescents participated. More adolescents than parents participated because some parents brought multiple children; however, to avoid clustering, only one adolescent per family was included in the analytic sample. When families had more than one adolescent present, an online randomizer was used to select one adolescent at random. The final analytic sample included 60 parent-adolescent dyads. Table 1 presents participant demographic characteristics. Adolescents ranged between 11 and 17 years old (*M* = 13.85 years, *SD* = 2.02) and were nearly evenly split by gender (53.3% female). Parents were 27 to 67 years old (*M* = 51.60, *SD* = 8.63). Most were biological mothers (77%), while 13.3% were other relatives (e.g., aunts, uncles, stepfathers, and sisters). Parents identified as Puerto Rican (43.3%), “Other” (18.3%), Mexican (13.3%), non-specified Hispanic/Latino (13.3%), and Dominican (11.7%). Among those identifying as “Other,” ethnic backgrounds included Central American (e.g., Guatemalan and Honduran), South American (e.g., Ecuadorian, Venezuelan, Peruvian), Dominican, and Black Latino.

87% of parents were born outside the U.S., almost double the rate of adolescents (43.3%). Most parents (58.3%) completed a high school degree or higher, and almost all (83.4%) expected their children to earn at least a bachelor’s degree. The median family annual income was approximately USD 20,000, lower than the median incomes for both the U.S. (USD 49,010) and state (USD 56,907) in 2016, though comparable to Puerto Rico’s median household income (USD 20,078; Guzman, 2017). Moreover, 36.45% of families lived at or below the household poverty line, and resided in moderately diverse neighborhoods, as indicated by an average Simpson’s Diversity Index of 0.60, a moderate level of diversity. The index ranges from 0 (no diversity) to 1 (maximum diversity) and measures the probability that two randomly selected individuals from a neighborhood will belong to different racial or ethnic groups. On average, neighborhood racial-ethnic composition included 39.55% Black and 25.54% Latine. Families resided in 14 different census tracts, and parents had lived in their present neighborhood for an average of 5.8 years (*SD* = 7.17).

### 2.2. Measures

#### 2.2.1. Residential Neighborhood Measures

**Neighborhood Connectedness.** Parents reported their perceptions of neighborhood social processes using ten items from Witherspoon and Hughes (2014), adapted from Perez-Smith et al. (2001) and Seidman et al. (1995). The scale assessed feelings of belonging and connection with neighbors (e.g., “I feel like I belong in my neighborhood”). One item was removed due to poor fit, resulting in a nine-item measure with high internal consistency (α = 0.92). Items were rated on a 4-point scale (1 = strongly disagree to 4 = strongly agree) and averaged to create a total score.

**Neighborhood Problems.** Parents completed sixteen items about physical (e.g., vandalism, property damage) and social (e.g., safety at night) problems within neighborhoods using the Neighborhood Problems Index (Perkins & Taylor, 1996). Items were rated on a 3-point Likert scale (1 = not a problem to 3 = a big problem) and averaged into a total score (α = 0.97).

#### 2.2.2. Socialization Measures

**Gender Role Socialization (GRS) Beliefs.** Parents’ GRS beliefs were measured using Hoffman and Kloska’s (1995) Gender-Based Attitudes Toward Child-Rearing (GATCR) subscale, which has demonstrated acceptable reliability in Latine samples (Adams et al., 2007) and in the current sample (α = 0.76). Four items assessed traditional beliefs about raising boys and girls differently (e.g., “It is more important to raise a son to be strong and independent than to raise a daughter that way”). Items were rated on a 4-point Likert scale (from 1 = strongly disagree to 4 = strongly agree) and averaged, with higher scores indicating more traditional beliefs.

**Ethnic–Racial Socialization (ERS) Practices.** Parents completed the 24-item ERS measure developed by Hughes and Johnson (2001), which assessed the frequency of three practices: cultural socialization, preparation for bias, and promotion of mistrust. Although ERS was initially conceptualized as a potential mediator, it was not included in the final models due to null associations with key variables and concerns about model saturation in this small pilot sample. This analytic decision is discussed in more detail in Appendix A, where we also report full-scale information and exploratory moderated mediation models to maintain transparency and inform future research.

#### 2.2.3. Adolescents’ Ethnic–Racial Identity (ERI)

Adolescents completed an adapted version of the Multidimensional Inventory of Black Identity (MIBI; Sellers et al., 1997), which has shown acceptable reliability with Latine youth (French & Chavez, 2010; Stein et al., 2017; Witherspoon et al., 2021). The adapted measure includes three subscales: centrality (e.g., “Being [my race/ethnicity] is an important reflection of who I am”), private regard (e.g., “I feel good about [my race/ethnicity] people), and public regard (e.g., “Others respect [my race/ethnicity] people). Adolescents rated each item on a 5-point Likert scale (1 = strongly disagree to 5 = strongly agree), and items were averaged within each subscale. Centrality originally included eight items; however, three reverse-coded items were omitted to improve internal consistency (α = 0.85) and because prior studies have shown that reverse-coded items often load poorly onto ERI factors, reduce validity, and confuse adolescent respondents—particularly in Spanish-speaking or Latine samples (Suárez Álvarez et al., 2018; Venta et al., 2022)^2^. Private regard included six items, with one reverse-coded item removed (α = 0.82). Public regard also included six items, with one reverse-coded item removed (α = 0.74).^3^

### 2.3. Analytic Plan

Hierarchical linear regressions were conducted to examine whether parents’ perceptions of their neighborhood and their GRS beliefs were associated with adolescents’ ERI outcomes: centrality, private regard, and public regard. GRS beliefs were tested as a potential moderator of the associations between neighborhood perceptions and ERI. Predictors were entered in four steps: (1) covariates (child ethnicity, child sex, and parent education), (2) neighborhood perceptions, (3) gender role socialization beliefs, and (4) the interaction between neighborhood perceptions and gender role beliefs. A total of six models were estimated: three models using neighborhood connectedness and three using neighborhood problems as predictors of the three ERI outcomes. All models tested GRS beliefs as a potential moderator and controlled for parent education, child ethnicity, and child gender. Child gender was tested during simple effects analyses of interactions.

ERS were initially conceptualized as a potential mediator between parents’ neighborhood perceptions and adolescents’ ERI outcomes; however, it was excluded from final models due to null associations with key study variables and concerns about model saturation in this small pilot study sample (*N* = 60). To preserve statistical power and reduce the risk of Type I and II errors (Babyak, 2004), the analytic plan was simplified from moderated mediation to moderation, focusing on the primary variables of interest: neighborhood context and GRS beliefs. Importantly, these null findings do not imply conceptual irrelevance—ERS may operate indirectly, interactively, or vary across ERI dimensions, as suggested in prior research (Umaña-Taylor & Hill, 2020; Ayón et al., 2020; Else-Quest & Morse, 2015; Sanchez et al., 2017; K. M. Schroeder & Bámaca-Colbert, 2019). For this reason, and to maintain transparency and inform future work, full measure details, reliability estimates, and exploratory moderated mediation models are provided in Appendix A.

Post hoc power analyses were conducted using G*Power 3.1 (Faul et al., 2007) to assess the ability to detect effects, including interactions, in regression models. With a sample size of 60 and α = 0.05, the study had 80% power to detect medium-sized effects (f^2^ ≥ 0.14) in models with up to 11 predictors. Additional post hoc analyses were not pursued, as prior work shows they do not reflect true power once results and correlations are known (Zhang et al., 2019). The small sample size limits the power to detect subtle effects, especially interactions. Non-significant *and* significant results should be interpreted with care. Still, by focusing on effect sizes and confidence intervals, this study aimed to offer a fuller view of the patterns observed, moving beyond reliance on *p*-values alone (Wilkinson, 1999).

### 2.4. Preliminary Analysis

Prior to conducting main analyses, regression assumptions were examined. Skewness and kurtosis values for all continuous variables fell within acceptable ranges. Visual inspection of residual plots indicated no major violations of linearity or homoscedasticity. Multicollinearity diagnostics also revealed no concerns, with all variance inflation factors (VIFs) below 1.26 and condition indices well below 30. Descriptive statistics and bivariate correlations for all study variables are presented in Table 2. ERS practices were not significantly correlated with neighborhood perceptions, GRS beliefs, or ERI outcomes, supporting their exclusion from the primary models.

On average, parents reported moderate neighborhood connectedness (*M* = 2.49, *SD* = 0.89) and problems (*M* = 1.74, *SD* = 0.72). Mean GRS beliefs were 1.50 (*SD* = 0.60), suggesting that most did not endorse traditional gender roles related to child-rearing practices. Independent samples *t-*tests showed no significant differences in GRS beliefs by adolescent gender *t*(58) = −1.52, *p* = 0.14. Adolescents reported relatively high levels of ERI centrality (*M* = 3.53, *SD* = 0.93), private regard (*M* = 4.14, *SD* = 0.65), and public regard (*M* = 3.28, *SD* = 0.72). These scores suggest that adolescents viewed their race and ethnicity as central to their self-concept, held positive views of their own racial-ethnic group, and perceived that others viewed their group favorably. No significant gender differences were found in centrality, *t*(58) = 0.53, *p* = 0.60, *d* = 0.01; private regard, *t*(58) = −0.28, *p* = 0.78, *d* = −0.07; or public regard, *t*(58) = 0.05, *p* = 0.96, *d* = 0.01.

## 3. Results

### 3.1. Neighborhood Connectedness, GRS Beliefs, and ERI Outcomes

To examine whether parents’ perceptions of neighborhood connectedness and gender role socialization (GRS) beliefs predicted adolescents’ ethnic–racial identity (ERI), a series of hierarchical linear regressions were conducted for each ERI outcome: centrality, private regard, and public regard. Each model included four blocks: (1) covariates (child ethnicity, child sex, parent education). (2) Neighborhood connectedness, (3) GRS beliefs, and (4) the interaction between connectedness and GRS beliefs. See Table 3 for model summaries.

**Model 1: ERI Centrality.** The final model was not significant, *F*(6, 53) = 1.16, *p* = 0.34, *R*^2^ = 0.12. Adding neighborhood connectedness did not improve model fit, Δ*R*^2^ = 0.01, Δ*F*(1, 55) = 0.33, *p* = 0.57, and neighborhood connectedness was not associated with centrality (*B* = −0.17, *p* = 0.14). GRS beliefs did not contribute significantly to the model, Δ*R*^2^ = 0.05, Δ*F*(1, 54) = 2.75, *p* = 0.10. The interaction between connectedness and GRS beliefs was also nonsignificant, *B* = 0.17, *p* = 0.49, Δ*R*^2^ = 0.01.

**Model 2: ERI Private Regard.** The final model was not significant, *F*(6, 53) = 1.09, *p* = 0.38, *R*^2^ = 0.11. Neighborhood connectedness marginally improved the model fit, Δ*R*^2^ = 0.047, Δ*F*(1, 55) = 2.88, *p* = 0.10, though it was not significantly associated with private regard (*B* = 0.12, *p* = 0.14). Neither GRS (*B* = 0.03, *p* = 0.67) nor the interaction term (*B* = 0.12, *p* = 0.47) was significant.

**Model 3: ERI Public Regard.** The full model was not significant, *F*(6, 53) = 1.64, *p* = 0.16, *R*^2^ = 0.16. No significant associations were found for neighborhood connectedness (*B* = 0.00, *p* = 0.97), GRS beliefs (*B* = 0.02, *p* = 0.55), or their interaction (*B* = 0.27, *p* = 0.15). However, parent education was negatively associated with public regard (*B* = −0.24, *p* = 0.02), such that higher parental education was linked to lower public regard.

### 3.2. Neighborhood Problems, GRS, and ERI Outcomes

Parallel hierarchical regressions were conducted using parents’ perceptions of neighborhood problems, GRS beliefs, and their interaction as predictors of ERI outcomes. (See Table 4 for model summaries and Table 5 for simple effects).

**Figure 2:**
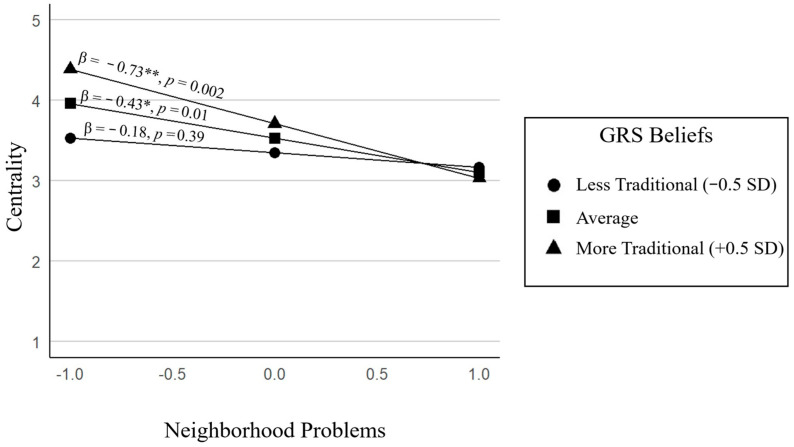
Moderating role of GRS on the association between neighborhood problems and centrality. * *p* < 0.05; ** *p* < 0.01.

**Model 4: ERI Centrality.** The final model significantly predicted ERI centrality, *F*(6, 53) = 3.08, *p* = 0.01, and explained 26% of the variance. The largest and most robust improvement in model fit occurred at Step 2, when neighborhood problems were added (Δ*R*^2^ = 0.09, Δ*F*(1, 55) = 5.80, *p* = 0.02). Higher levels of perceived neighborhood problems were associated with lower ERI centrality (*B* = −0.43, *p* = 0.01). More traditional GRS beliefs were associated with higher centrality (*B* = 0.38, *p* = 0.01, Δ*R*^2^ = 0.06), and the interaction term was marginally significant (*B* = −0.50, *p* = 0.06, Δ*R*^2^ = 0.05), suggesting a potential moderating effect. (See Figure 2). Among adolescents whose parents endorsed more traditional GRS beliefs (+0.5 *SD*), higher neighborhood problems were associated with lower ERI centrality. In contrast, when parents held less traditional beliefs (−0.5 *SD*), neighborhood problems were not significantly related to ERI centrality. These findings suggest that, in the context of traditional gender expectations, adolescents living in neighborhoods perceived by their parents as higher in risk may experience fewer opportunities or supports for engaging with their racial and ethnic identity beyond their home. Conversely, parents who hold more egalitarian gender role beliefs may be more likely to encourage youth’s identity development even in the face of neighborhood problems.

**Figure 3:**
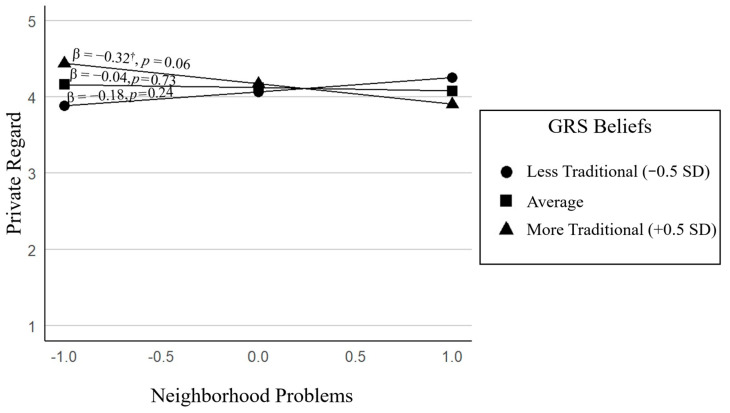
Moderating role of GRS on the association between neighborhood problems and private regard. ^†^ *p* < 0.06.

**Model 5: ERI Private Regard.** The full model was not significant, *F*(6, 53) = 1.65, *p* = 0.15. However, the interaction between neighborhood problems and GRS significantly improved model fit, Δ*R*^2^ = 0.09, Δ*F*(1, 53) = 5.80, *p* = 0.02, and was associated with private regard, *B* = −0.45, *p = 0*.02, indicating that more perceived neighborhood problems were associated with lower private regard among adolescents whose parents endorsed more traditional GRS beliefs (+0.5 *SD*), suggesting that in the context of traditional GRS beliefs, neighborhood risk may undermine how positively youth feel about their ethnic–racial group. In contrast, when parents held less traditional beliefs (−0.5 *SD*), neighborhood problems were not significantly related to private regard, indicating that more egalitarian beliefs may buffer against the negative effects of neighborhood risk on adolescents’ ethnic–racial self-perceptions (See Figure 3). No other predictors in the model were significantly associated with private regard.

**Figure 4:**
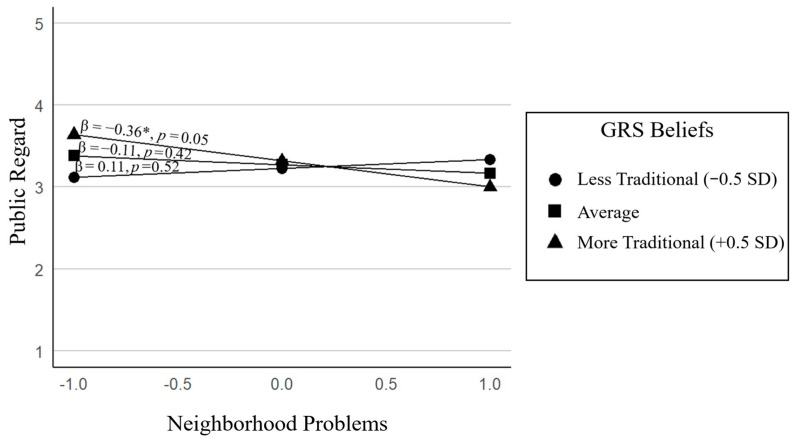
Moderating role of GRS on the association between neighborhood problems and public regard. * *p* < 0.05.

**Model 6: ERI Public Regard.** The full model approached significance, *F*(6, 53) = 2.19, *p* = 0.06, *R*^2^ = 0.20. Adding the interaction term significantly improved model fit, Δ*R*^2^ = 0.07, Δ*F*(1, 53) = 4.42, *p* = 0.04. GRS modified the association between neighborhood problems and public regard, *B* = −0.43, *p* = 0.04, such that more neighborhood problems predicted lower public regard, but only among adolescents whose parents endorsed more traditional GRS beliefs (+0.5 *SD*). That is, when parents held more traditional GRS beliefs, adolescents in higher-risk neighborhoods perceived worse societal views of their ethnic–racial group (lower public regard). When parents held less traditional beliefs, neighborhood problems did not significantly relate to public regard (see Figure 4). This suggests that traditional gender expectations may heighten adolescents’ sensitivity to contextual risks, such as neighborhood problems, shaping how they perceive their group is viewed by society. In contrast, more egalitarian beliefs may buffer adolescents from internalizing negative societal messages in the face of contextual stressors. Additionally, parent education was negatively associated with public regard (*B* = −0.24, *p* = 0.01), such that adolescents of more highly educated parents perceived more negative societal views of their ethnic–racial group.

## 4. Discussion

This exploratory pilot study examined how Latine parents’ neighborhood perceptions, GRS beliefs, and ERS practices related to adolescents’ ERI in a new destination context. Specifically, we tested whether GRS beliefs moderated associations between neighborhood perceptions of connectedness and problems and ERI. Our hypotheses were partially supported.

### 4.1. Neighborhood Effects on ERI

We first hypothesized that more neighborhood connectedness would be associated with stronger ERI (centrality and private regard), whereas more neighborhood problems would be linked to lower ERI. This hypothesis was only partially supported. Contrary to expectations, neighborhood connectedness was not significantly associated with any ERI outcome. However, perceptions of neighborhood problems were negatively associated with ERI centrality, though not with public or private regard. Adolescents whose parents perceived more neighborhood problems reported placing less importance on their ethnic–racial identity compared to peers in less problematic environments.

This finding aligns with ecological and sociocultural models of development (García Coll et al., 1996; Witherspoon et al., 2023), which posit that proximal environmental stressors—like neighborhood disorganization (Shaw & McKay, 1942), including perceived risk—can undermine adolescents’ identity development. Specifically, perceptions of neighborhood risk may constrain opportunities for exploration, cultural affirmation, or safe group affiliation, thereby lowering the salience of ERI centrality. Social disorganization theory similarly suggests that higher neighborhood disorder (e.g., poverty, residential instability, ethnic heterogeneity) may erode community-level support systems and social cohesion, which are important resources for positive identity development (Witherspoon et al., 2023).

Prior empirical work has also shown that neighborhood risk is associated with lower ERI centrality and exploration, particularly among Latine youth (Rivas-Drake, 2012), and that such risks may indirectly shape identity through family processes like ERS (Lambert et al., 2021). More broadly, studies with immigrant-origin adolescents have found that neighborhood violence and social disorder are related to weakened cultural identity by limiting access to supportive community spaces (White et al., 2018). Together, the current study supports findings that suggest that ERI centrality may be especially sensitive to contextual risk, such as perceived threats. Regard is a more affective dimension of ERI and may be more strongly shaped by distal or relational factors, including school climate, peer dynamics, and media exposure (Neblett et al., 2009). Thus, while perceptions of neighborhood problems may undermine the perceived importance of ERI, other aspects of identity may be less directly impacted by local environmental stressors.

Still, the lack of association between neighborhood connectedness and ERI was unexpected, given prior work linking community support to positive youth outcomes. One possible explanation is a ceiling effect: parents in this sample reported relatively high neighborhood connectedness, which may have limited variability and statistical power, especially given the sample size. Additionally, connectedness may be less salient than neighborhood problems for shaping identity in new destination contexts, where Latine families may lean more on intra-familial and cultural networks than on broader neighborhood relationships (Ayón et al., 2020; Cahill et al., 2021).

Contextual factors also likely contributed to this pattern. The present study took place in a diverse, lower-income Northeastern city with limited Latine-serving infrastructure—despite a 56% growth in the Latine population between 2000 and 2010 (Pew Research Center, 2008). Only one cultural center served the entire community at the time of data collection in 2016. The limited institutional resources, which are often a characteristic of new destination contexts, likely make it difficult for some residents to establish connections and social ties and reap the benefits of such capital for their own development and wellbeing.

It is also possible that neighborhood connectedness functions differently depending on the broader structural context. Prior work suggests it may only serve a protective role when structural risks are low or otherwise accounted for (Oyserman & Yoon, 2009; Martinez & Polo, 2018) and that it may vary in its impact across racial and ethnic groups (Hull et al., 2008). Because this study modeled neighborhood connectedness and problems separately, we were unable to assess their interactive effects. Future research should examine how social resources and structural risks interact, and whether unmeasured aspects of connectedness better explain identity outcomes. More nuanced qualitative interviews with families in new destination contexts may help reveal what they consider a connected neighborhood to be in a new destination context, what they identify as sources of support or disconnection, and how these experiences relate to adolescents’ ERI development.

In addition to these structural and contextual explanations, the broader sociopolitical climate at the time of data collection in 2016 may have also heightened parents’ sensitivity to problems versus connectedness in their neighborhoods. The 2016 election season between Donald Trump and Hillary Clinton was marked by heightened national anti-immigration rhetoric that erased distinctions among Latine subgroups and fueled a generalized climate of fear, marginalization, and identity threat. Although limited, research on adolescents’ responses during this election found that Latine adolescents reported heightened emotional distress, identity threat, and increased vigilance in response to anti-immigrant sentiment—including avoiding certain places or people, or adjusting their behavior to reduce perceived risk, including avoiding speaking Spanish in public during the 2016 election season (Wray-Lake et al., 2018; DeJonckheere et al., 2018; Philbin & Ayón, 2016). Likewise, Lorenzo-Blanco et al. (2019) collected data starting in 2016 and found that cultural stressors—such as discrimination and negative context of reception—predicted poorer adolescent adjustment, particularly in neighborhoods with more perceived problems. Other research has found that in the context of heightened anti-immigrant rhetoric in 2016, Latine immigrant parents became more attuned to potential threats and responded with protective strategies aimed at safeguarding their children (Philbin & Ayón, 2016), over cultivating broader community connectedness. Thus, parents’ perceptions of more neighborhood problems, over-connectedness, may be a result of sociopolitical threat and stress from the 2016 presidential election season.^4^

Finally, we wonder whether adolescents’ perceptions of neighborhood connectedness would have emerged as a stronger predictor of their own ERI development. Prior work suggests that adolescents’ own sense of neighborhood may be more proximally predictive of development outcomes (Witherspoon et al., 2023; Witherspoon & Hughes, 2014), especially as they gain independence and spend more time outside of their parents’ gaze with peers. Including adolescents’ own experiences of neighborhood would also allow for direct comparisons to parents experiences of neighborhood to determine (1) whether perspectives differ from between adolescents and parents in this context; (2) whether adolescents’ perspectives have a different direction or effect size on ERI development; and (3) how parent and adolescents’ neighborhood perceptions work each uniquely and together shape ERI development (e.g., Witherspoon & Ennett, 2011). It is also important to note that any generational or acculturative differences in neighborhood perception between adolescents and parents could have been amplified by the sociopolitical climate of 2016. This distinction may be particularly relevant for Puerto Rican families in our sample, where approximately 87% of parents were born outside the continental U.S. whereas about 56.6% of adolescents were born within it.

### 4.2. GRS Beliefs as a Moderator

Another goal of this study was to examine how GRS beliefs shape the impact of neighborhood context on ERI. Our second hypothesis expected that adolescents whose parents endorsed more traditional GRS beliefs would report lower ERI outcomes in the context of high neighborhood problems, whereas those with more egalitarian parents in a similar neighborhood context would have higher ERI outcomes.

This hypothesis was partially supported. GRS beliefs did not moderate the relation between neighborhood connectedness and ERI. However, they did moderate the association of neighborhood problems with centrality, private regard, and public regard. Specifically, greater perceived neighborhood problems were associated with lower ERI outcomes, particularly among adolescents whose parents held more traditional GRS beliefs. For ERI centrality, this negative association was also significant among adolescents whose parents held average GRS beliefs. No associations were found for adolescents with egalitarian parents. Notably, gender did not moderate these associations: the effects of traditional GRS beliefs and neighborhood problems were similar for boys and girls, suggesting that marianismo, machismo, and other culturally rooted parenting frameworks may operate similarly across genders, particularly in contexts where parents perceive elevated neighborhood risk.

These findings align with cultural-ecological and intersectional theories of development, which emphasize that youth identity processes are shaped not only by environmental risks but also by the cultural values and belief systems that families use to interpret and respond to those risks (Witherspoon et al., 2023; García Coll et al., 1996) For Latine families, gender role beliefs rooted in marianismo and machismo may function as protective parenting strategies—culturally informed expectations about appropriate behavior that guide decisions about youth autonomy, mobility, and exposure to external contexts, especially when environments are perceived as unsafe (Raffaelli & Ontai, 2004; K. M. Schroeder & Bámaca-Colbert, 2019; Sanchez et al., 2016). In such cases, more traditional beliefs may restrict opportunities for exploration or social interaction, both of which are central to ERI development (Umaña-Taylor et al., 2014).

At the same time, the presence of these patterns across both girls and boys suggests that gendered cultural expectations—though often differentially imposed—shape identity development for all youth, challenging the assumption that only girls are impacted by traditional GRS (Raffaelli & Ontai, 2004; Gallegos-Castillo, 2006). Boys, too, are affected in both positive and negative ways by patriarchal cultural norms, such as the contrasting expectations of *machismo* and *caballerismo* (Rivera et al., 2024; Arciniega et al., 2008; DeGue et al., 2024). In other words, cultural scripts around gender are not inherently “good” or “bad,” but are complex, context-dependent strategies that families may draw upon to navigate risk, safety, and belonging.

Although we expected to find gender differences, the absence of such effects may reflect shared vulnerability across gender in high-risk environments within new destinations for Latine families, where both girls and boys are exposed to similar structural stressors and protective family factors. Alternatively, this may be due to sample size and measurement limitations—such as relying on binary sex as a proxy for gender (which reflects a bigger pattern in developmental science conflating sex and gender^5^)—or social desirability in parent-reports of GRS use. Longitudinal and qualitative methods would be especially useful for capturing how gendered cultural values and socialization intersect with context to shape identity development over time (e.g., developmental periods, sociohistorical contexts) and across place. Future research should also examine gender socialization and gender beliefs as an adaptive culture—belief and practice—that may vary across diverse ecological contexts (White et al., 2018). This work must also move beyond binary frameworks that consider how cultural values shape development for all youth—including gender-diverse youth—in dynamic, situated ways (Witherspoon et al., 2023; Garíca Coll et al., 1996).

### 4.3. GRS Beliefs Vs. ERS Practices and Null Effects

While the study’s primary focus was on parents’ GRS beliefs and neighborhood context, ERS was initially conceptualized as a potential mediator through which parents’ perceptions of neighborhood conditions would shape adolescents’ ERI content. We hypothesized that parents would adjust their ERS practices in response to environmental cues, with the strength and direction of these effects varying by GRS beliefs (see Appendix A). However, ERS was not significantly associated with neighborhood variables, GRS beliefs, or ERI outcomes. Including ERS in the model oversaturated the analysis and reduced power in this small pilot sample (*N* = 60), prompting us to simplify the analytic plan from moderated mediation to moderation, particularly given our primary interest in GRS beliefs over ERS practices. This adjustment was made prior to final model testing and allowed for a more focused investigation of how GRS beliefs may shape the meaning and impact of parents’ neighborhood perceptions on adolescents’ ERI.

To clarify that these null findings likely reflect analytic and contextual constraints rather than conceptual irrelevance, all ERS-related analyses are reported in Appendix A and discussed below. To that end, we offer several theoretically grounded reasons why ERS practices may not have emerged as significant in this sample, despite their well-established role in the broader literature.

There are several reasons ERS practices may not have emerged as significant in this study. First, prior work suggests that the relation between neighborhood context and ERS is often conditional on other factors, such as parent ethnic-racial identity, perceived safety, or contextual stressors. In a new destination context especially, there is still a lot to learn about the unique processes and adaptations parents make in this new context. For example, analyses using the same dataset found that cultural socialization was only associated with neighborhood context when moderated by perceptions of safety or racial diversity (Witherspoon et al., 2021). These findings suggest that ERS may not have consistent direct associations with neighborhood variables or certain adolescent outcomes, and may instead function as part of more complex, context-dependent processes and explain why, in the present study, ERS was not associated with other study concepts.

The question remains: why did GRS beliefs—but not ERS practices—show significant associations with neighborhood conditions and ERI outcomes in this sample? One possibility is that beliefs and practices reflect different dimensions of parenting. Beliefs, particularly those related to gender roles and cultural expectations, may be closely tied to parents’ self-efficacy—that is, their confidence in navigating their environment and making decisions that protect and guide their children (Witherspoon et al., 2023; García Coll et al., 1996; White et al., 2018; Ardelt & Eccles, 2001). Practices, by contrast, reflect behavioral competency or enacted strategies that may be more routinized and less immediately responsive to shifting contextual cues. This distinction may be particularly relevant in new destinations, where families may be adapting to unfamiliar social landscapes and navigating environments with fewer culturally familiar supports or institutions (Stein et al., 2016; Marrow, 2020; Witherspoon et al., 2021; Lambert et al., 2021).

In contrast, GRS beliefs—rooted in gendered cultural values—may more directly reflect how parents interpret and regulate engagement with their neighborhoods, especially in new destination settings where co-ethnic resources are less visible. ERS practices, however, may be more consistent and guided by cultural tradition, and thus less sensitive to day-to-day environmental variation. GRS beliefs may be especially sensitive to how parents interpret neighborhood risks, particularly in under-resourced or unfamiliar contexts like new destination communities (Witherspoon et al., 2023; Stein et al., 2016). Parents may rely on gendered cultural values to guide decisions about their children’s autonomy and exposure to perceived threats (Raffaelli & Ontai, 2004; K. M. Schroeder & Bámaca-Colbert, 2019; Sanchez et al., 2016).

Another possible explanation is that our operationalization of ERS—based on the frequency of cultural socialization, preparation for bias, and promotion of mistrust—may not align with Latine parents’ culturally grounded socialization goals (Stein et al., 2016; Hughes et al., 2006). Recent reviews point out that Latine parents use preparation for bias and promotion of mistrust less frequently than cultural socialization, potentially due to discomfort conveying mistrust or negative emotions (Ayón et al., 2020; Cross, 2022; Perez-Brena et al., 2024). These practices may reflect broader cultural norms and vary depending on immigrant status, for example, rather than reacting to neighborhood context or perceived risks.

### 4.4. Limitations and Future Directions

This exploratory pilot study focused on how parental gender role socialization (GRS) beliefs and neighborhood perceptions related to Latine adolescents’ ERI content. In addition to those mentioned already in the *Discussion*, we highlight several limitations that warrant future research.

First, longitudinal and observational work is needed to capture the dynamic nature of GRS and ERS together and over time, particularly in new destination contexts. Prior conceptual work underscores how gender, race, and ethnicity are deeply intertwined, and how GRS and ERS together shape adolescents’ sense of self (and wellbeing) depending on where they find themselves. For example, work with Mexican-heritage adolescents (ages 12–14) living in the Southwest U.S. found that girls often received more restrictive parenting and appearance-focused parenting messages than boys (Raffaelli & Ontai, 2004). Similarly, interviews with adult Latina women living in the Northeastern highlight how tensions around skin tone, language, and appearance shaped both their adolescent ERI development and their views of their parents’ ERI (Araujo Dawson & Quiros, 2014). Not speaking Spanish, having darker skin, or “not looking Latina enough” contributed to exclusion and heightened exposure to ERS messages such as preparation for bias or promotion of mistrust—messages less commonly reported in Latine families more broadly. These findings align with research on colorism and racial identity among Latinas (Quiros & Dawson, 2013). Relatedly, a study with 198 rural Latine youth found that neighborhood risk and discrimination were linked to higher levels of depressive symptoms—particularly among girls—suggesting that Latina adolescents may face gendered ethnic–racial discrimination, prompting increased parental behavioral monitoring and relational control (Bermudez et al., 2025). While ERI affirmation helped buffer these effects, ERI exploration sometimes heightened distress, especially in high-risk neighborhoods. Future work should explore how ERI may not only be shaped by socialization and context but also function as a moderator or mediator in how parenting and neighborhood environments shape youth experiences across different places.

Secondly, the generalizability of our findings is limited to the context and sample of this pilot study, primarily comprising Puerto Rican youth and their mothers residing in a new destination—a historically Black city in Pennsylvania. Puerto Ricans occupy a unique sociopolitical position in the U.S.: although citizens by birth, they are often misrecognized and subjected to anti-immigrant discrimination (Wray-Lake et al., 2018). As Capielo Rosario et al. (2023) contend, most psychological models assume voluntary migration and fail to account for the complexity of Puerto Rican migration, which is more often shaped by colonial displacement, economic instability, and structural neglect by the U.S. than by the voluntary movement assumed in most psychological models. Phrases like “It hurts, but it’s the thing we do” (Gonzalez et al., 2021) or “Half here, half there” (Guzzardo et al., 2016), offer insight into how Puerto Rican families make meaning of migration, acculturation, community, and identity in ways that may differ from other Latine subgroups.

Within this Puerto Rican subgroup, our data largely reflects mothers’ perspectives (76.7% of the sample). Mothers play crucial roles in shaping youth identity (Umaña-Taylor & Bámaca, 2004) as both cultural transmitters and protectors, especially in the face of migration-related stress. The unique responsibilities mothers carry may shape their perceptions of neighborhood and socialization beliefs, around both ethnicity, race, and gender, that differ from those of other family members, including fathers, grandparents, or siblings. Multigenerational or mixed-status households may also express different, and perhaps conflicting, gendered messages, possibly shaped by neighborhood concerns (K. M. Schroeder & Bámaca-Colbert, 2019; Hurtado, 2003; Umaña-Taylor & Bámaca, 2004). Future work with a larger, more diverse sample of parents may be needed to understand how family structure shapes socialization processes and ERI development.

Finally, the timing of our data collection—2016—raises important questions about generalizability. Although the data are nearly a decade old, the same anti-immigrant rhetoric that characterized the highly polarizing U.S. presidential election in 2016 set the stage for that of 2024. In fact, Trump’s second administration has enforced even stricter anti-immigration policies and rhetoric (National Immigration Forum, 2025; Brookings Institution, 2025). As discussed in Section 4.1, perceptions of more neighborhood problems may have been more strongly influenced than connectedness by heightened fear, misrecognition, and intergroup tensions in the wake of the 2016 election (Wray-Lake et al., 2018; Lopez et al., 2018), which is likely still relevant today—if not more so.

Specifically, Vance’s rhetoric about the rural South and Appalachia (e.g., McCarroll & Harkins, 2019), combined with his hardline stance on immigration, may profoundly shape how Latine youth in 2025 relate to their heritage and regard themselves and other members of their racial-ethnic group, particularly as more Latine immigrant families settle in new *rural* destinations (Lichter, 2012; Stein et al., 2016). Future research could examine whether the patterns observed in this exploratory pilot study would replicate in a more recent sample of Latine youth in similar new destination contexts. Might these effects persist—or intensify—under current sociopolitical conditions? At the same time, discrimination may prompt stronger identification with one’s marginalized group, as individuals seek belonging and affirmation (Lorenzo-Blanco et al., 2019). In such cases, perceptions of neighborhood connectedness might actually increase depending on who is reporting (parents vs. youth) and the characteristics of the new destination (e.g., rural vs. urban; racial-ethnic heterogeneity). As the U.S. continues to diversify amidst increasingly divisive and extreme sociopolitical ideologies around race, gender, and immigration, it is even more urgent to understand the conditions under which Latine youth are able to grow into themselves (i.e., ERI development)—and how parents and communities can support that growth.

## 5. Conclusions

Grounded by cultural- and contextual-developmental theories, the current exploratory pilot study sought to understand the lived experiences of Latine families in a new destination context, with specific attention to how Latine youth’s ERI are impacted by neighborhood social dynamics and culture-related parenting. GRS emerged as a meaningful contributor to this developmental process, especially in neighborhoods viewed as riskier or less safe. With current demographic shifts and U.S. stratification and structural discrimination, Latine families are more likely to reside in lower-income neighborhoods, which may contribute to additional concerns for parents. Our work suggests that in such neighborhoods, and especially ones where there may be fewer co-ethnics (i.e., new destinations), parents might benefit from neighborhood and/or parenting interventions that include a focus on both environmental and parental practices and beliefs.

Interventions such as *Your Family, Your Neighborhoods* (Brisson et al., 2019; Lechuga-Peña et al., 2020) could be adapted to not only include neighborhood cohesion and trust (akin to connectedness) but also include psychoeducational elements related to GRS. Further, it is important to note that these complex relations were most impactful for regard—how youth feel about their own ethnic–racial group (i.e., private regard) and how youth perceive others view their ethnic–racial group (i.e., public regard). These findings demonstrate the critical need for universal ERI and ERS interventions—such as the *Identity Project* (Umaña-Taylor et al., 2018) and *One Talk at a Time* (Stein et al., 2021)—that adhere to anti-racist and anti-xenophobia frameworks to support youth as they navigate and understand their identity in relation to the world around them. Though our study did not identify ERS as a major contributor to ERI, the ways in which parents’ gender role beliefs worked, in concert with perceptions of neighborhood risk to impact ERI, suggest that interventions that focus on helping families of color have conversations about ethnicity-race, and what it means for their families in the contexts they are embedded, are critically necessary and must include attention to gendered roles and scripts.

## Figures and Tables

**Table 1:** Participant demographic characteristics.

		Parents (*N* = 60)	Adolescents (*N* = 60)
Age		41.06 (9.52)	13.85 (2.02)
Years living in current neighborhood		5.8 (7.17)	-
Ethnicity			
	Puerto Rican	26 (43.3%)	24 (40%)
	Dominican	7 (11.7%)	8 (13.3%)
	Mexican	8 (13.3%)	7 (11.7%)
	Hispanic/Latino/Americana	8 (13.3%)	8 (13.3%)
	Other	11 (18.3%)	13 (21.67%)
Adolescent Sex			
	Male	-	28 (46.7%)
	Female	-	32 (53.3%)
Relation of parent to child			
	Mother	46 (76.7%)	-
	Father	6 (10.0%)	-
	Aunt	2 (3.3%)	-
	Grandmother	2 (3.3%)	-
	Other	4 (6.7%)	-
Parent Education			
	Less than high school	25 (41.7%)	-
	High school or equivalent	14 (23.3%)	-
	Some post-secondary education	17 (28.3%)	-
	Bachelor’s degree	4 (6.7%)	-
Marriage/cohabitation status			
	Married or cohabitating	29 (48.3)%	-
	Not Married or cohabitating	18 (30.0%)	-
	Divorced or separated	13 (12.7%)	-
Nativity			
	Born outside U.S. 50 states	52 (86.67%)	26 (43.3%)
	Born in the U.S.	8 (13.33%)	34 (56.6%)

Note. Other ethnicities reported include Dominican, Peruvian, Guatemalan, Ecuadorian, Venezuelan, Black Latino, Honduran, Chicana, Salvadorian, and American.

**Table 2:** Means, standard deviations, and bivariate correlations among study variables.

Variables	1.	2.	3.	4.	5.	6.	7.	8.	9.	10.
1. Parent Education	-									
2. RN Connectedness	−0.12	-								
3. RN Problems	−0.02	−0.40 **	-							
4. Cultural Socialization (CS)	0.32 *	0.01	−0.00	-						
5. Prep for Bias (PB)	0.16	0.02	0.25 ^†^	0.49 ***	-					
6. Promotion of Mistrust (PMT)	−0.09	0.17	0.19	0.12	0.35 **	-				
7. Gender Role Socialization (GRS)	−0.08	0.26 *	−0.01	−0.14	0.10	0.27 *	-			
8. Centrality	−0.19	0.10	−00.2	0.11	−0.12	0.02	0.22	-		
9. Private Regard	−0.21	0.24 ^†^	−0.04	−0.08	−0.16	0.11	0.12	0.64 ***	-	
10. Public Regard	−0.33 *	0.08	−0.06	−0.00	−0.16	0.11	0.09	0.60 ***	0.61 ***	-
*N*	60	60	60	60	60	60	60	60	60	60
M	2.00	2.49	1.74	3.67	3.35	1.90	1.50	3.53	4.14	3.28
*SD*	0.99	0.89	0.72	0.87	0.81	0.82	0.60	0.93	0.65	0.72
Min	1.00	1.00	1.00	1.43	1.33	1.00	1.00	1.40	2.40	1.20
Max	4.00	4.00	3.00	5.00	5.00	5.00	3.00	5.00	5.00	5.00
Skewness	-	−0.03	0.52	−0.39	−0.20	1.78	0.96	−0.16	−0.40	0.09
Kurtosis	-	−1.17	−1.35	−0.63	−0.50	4.09	−0.11	−0.58	−0.44	0.42

Notes. ^†^ marginally significant, *p* < 0.06; * *p* < 0.05; ** *p* < 0.01; *** *p* < 0.001 (all two-tailed).

**Table 3:** Final hierarchical linear regression models predicting ERI outcomes from neighborhood connectedness.

	Parameter Estimates and *p* Values		
	Model 1	Model 2	Model 3
Variables	Centrality	Private Regard	Public Regard
(Intercept)	4.00 (0.34) ***	4.33 (0.24) ***	3.76 (0.25) ***
Neighborhood Connectedness	−0.17 (0.14)	0.12 (0.14)	0.12 (0.14)
Gender Role Socialization (GRS)	0.30 (0.22)	0.03 (0.15)	0.02 (0.17)
Neighborhood Connectedness × GRS	−0.17 (0.25)	0.12 (0.17)	0.27 (0.18)
Child Gender	−0.32 (0.25)	−0.05 (0.18)	−0.17 (0.19)
Child Ethnicity	0.15 (0.25)	0.11 (0.17)	0.09 (0.18)
Parent Education	−0.20 (0.13)	−0.12 (0.09)	−0.24 (0.10) *
*N*	60	60	60
*R* ^2^	0.18	0.11	0.16
adj. *R*^2^	0.02	0.01	0.06
Resid. sd	0.85	0.64	0.69

Note. Unstandardized regression coefficients (B) are reported. Standard errors are in parentheses. Significant at * *p* < 0.05; *** *p* < 0.001.

**Table 4:** Final hierarchical linear regression models predicting ERI outcomes from neighborhood problems.

	Parameter Estimates and *p* Values		
	Model 4	Model 5	Model 6
Variables	Centrality	Private Regard	Public Regard
(Intercept)	4.23 (0.31) ***	4.41 (0.23) ***	3.88 (0.25) ***
Neighborhood Problems	−0.43 (0.16) *	−0.04 (0.12)	−0.11 (0.13)
Gender Role Socialization (GRS)	0.36 (0.19) ^†^	0.10 (0.14)	0.09 (0.15)
Neighborhood Problems × GRS	−0.50 (0.26) ^†^	−0.45 (0.19) *	−0.43 (0.20) *
Child Gender	−0.57 (0.25) *	−0.10 (0.18)	−0.23 (0.20)
Child Ethnicity	0.00 (0.23)	0.03 (0.17)	−0.03 (0.18)
Parent Education	−0.22 (0.12) ^†^	−0.13 (0.09)	−0.24 (0.09) *
*N*	60	60	60
*R* ^2^	0.51	0.16	0.20
adj. *R*^2^	0.18	0.06	0.11
Resid. sd	0.85	0.63	0.68

Note. Unstandardized regression coefficients (B) are reported. Standard errors are in parentheses. ^†^ *p* < 0.06. * *p* < 0.05. *** *p* < 0.001.

**Table 5:** Simple effects of neighborhood problems on ERI by GRS beliefs.

Outcome	GRS Level	β	*SE*	95% CI
Centrality	−0.50	−0.18	0.21	[−0.60, 0.24]
	0	−0.43 *	0.16	[−0.76, −0.10]
	+0.60	−0.73 **	0.22	[−1.18, −0.28]
Private Regard	−0.50	0.19	0.15	[−0.12, 0.50]
	0	−0.04	0.12	[−0.28, 0.20]
	+0.60	−0.32 ^†^	0.17	[−0.65, 0.01]
Public Regard	−0.50	0.11	0.17	[−0.23, 0.44]
	0	−0.11	0.13	[−0.37, 0.16]
	+0.60	−0.36 ^†^	0.18	[−0.72, −0.01]

Note. Unstandardized regression coefficients (B) are reported. ^†^ *p* < 0.06. * *p* < 0.05. ** *p* < 0.01.

**Figure A2:**
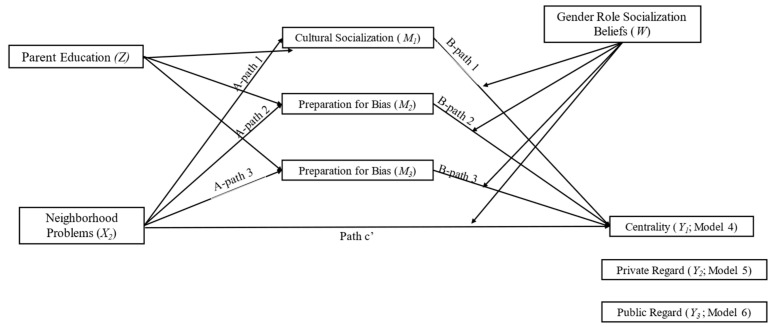
Conceptual moderated mediation model. Neighborhood problems predicting ERI (i.e., *centrality*—Model 4; private regard—Model 5; public regard—Model 6) through ERS, moderated by GRS.

## Data Availability

The datasets presented in this article are not readily available because of the sensitivity of the study data, sample, and context, and because we did not obtain IRB approval to share the data externally. Additionally, data analyses are actively ongoing as part of a larger project. Further inquiries can be directed to the corresponding author.

## Notes

1 The gender-neutral term *Latine* is used in the current study for two reasons. First, the current study sample did not require adolescents or parents to identify as binary gender identities (i.e., Latino/a). Second, the term *Latine* aligns better with the grammatical structure of the Spanish language and has been increasingly used by Spanish speakers and younger generations as a more accessible alternative to *Latinx* (Miranda et al., 2023).

2 To ensure that the exclusion of reverse-coded items did not artificially inflate reliability or change our interpretation of results, we reran analyses (i.e., Models 1–6) using the full original subscales, including reverse-coded items. The overall patterns of results remained consistent, including the key interaction term between neighborhood problems and GRS. Full results from these robustness checks are available upon request.

3 The same participants missing data for the private regard subscale also were missing data for the public regard subscale and again replaced with the sample subscale mean (i.e., *M* = 3.28, *SD* = 0.72).

4 We do not conceptualize neighborhood problems (or connectedness) as proxies for American culture. Instead, neighborhood context is a proximal development setting that may intersect with, but remains distinct from, broader societal ideologies and family-held cultural beliefs [Witherspoon et al., 2023]. These exploratory pilot findings reflect the interaction of multiple, layered influences on adolescent development in new destination contexts.

5 For a comprehensive history of how gender and sex have been conceptually, empirically, and ontologically conflated—especially in relation to race—in child development fields, see *How the Clinic Made Gender* (Eder, 2022).

## Appendix A. Parent-Reported ERS Measures and Exploratory Moderated Mediation Analysis Details

Mediation analyses tested the hypothesis that parents’ perception of neighborhood may impact adolescents’ ERI indirectly through their socialization practices around ethnicity and race. One moderation was added to test whether parents’ adherence to traditional gender role beliefs (i.e., raising boys and girls differently to fulfill traditional gender roles) changes the relation between ethnic–racial socialization and adolescents’ ethnic–racial identity. Moderated mediation analyses were conducted using Model 15 of Hayes’ PROCESS Macro software (Version 4.2; Hayes, 2017), which allowed for a more comprehensive approach to exploring potential moderating effects. Model 15 was intentionally selected over Model 14 and other PROCESS models because it examines moderating effects on both the B path and C’ prime path. In contrast, Model 14 only examines moderating effects on the B path. Bootstrap interference was used to avoid possible non-normalities in the A or B paths.

Exploratory moderated mediation analyses were initially conducted using Hayes’ PROCESS Macro (Model 15) with 10,000 bootstrapped samples and HC3 robust standard errors. Models tested whether gender role socialization (GRS) moderated associations between parents’ neighborhood perceptions (connectedness, problems) and adolescents’ ERI (centrality, private regard, public regard), both directly and indirectly via ERS practices. (See Conceptual Models in Figure A1 and Figure A2). Parent education was included as a covariate; adolescent gender and race/ethnicity were excluded to preserve power. Although few significant indirect effects were observed, multiple interactions between GRS and neighborhood problems emerged, suggesting that gendered parenting beliefs may shape the influence of contextual risk on adolescents’ identity development.

PROCESS models are based on regressions but do not check assumptions (i.e., of normality, homoscedasticity, linearity). To control for normality, all regression coefficients were bootstrapped (10,000 resamples). To control for homoscedasticity and adjust for small sample size, robust standard errors (HC3) were used. Assumptions of linearity were assessed using simple scatterplots. Skew and kurtosis were checked at both item and measure levels, as was measure internal consistency. Results are presented in Table A1 and Table A2.

**Table A1:** Regression coefficients for neighborhood connectedness models 1–3.

Neighborhood Connectedness Models (1–3)										
		Consequent								
		(a-path is the same for models 1–3)						**Model 1**	**Model 2**	**Model 3**
		*M*_1_ (CS)		*M*_2_ (PB)		*M*_3_ (PMT)		*Y*_1_ (Centrality)	*Y*_2_ (Private Regard)	*Y*_3_ (Public Regard)
Antecedent		Coeff. (*SE*)		Coeff. (*SE*)		Coeff. (*SE*)		Coeff. (*SE*)	Coeff. (*SE*)	Coeff. (*SE*)
Constant	*i*_1_	−0.57 * (0.25)		−0.27 (0.29)		0.17 (0.27)		0.33 (0.58)	40.31 (0.39) ***	0.46 (0.55)
X (RN Connectedness)	*a*_1_	0.05 (0.12)		0.03 (0.12)		0.18 (0.13)	*c*’	0.03 (0.17)	0.21 (0.11)	0.06 (0.13)
Z (Parent Education)	*a*_2_	0.28 ** (0.10)		0.14 (0.12)		0.19 (0.11)		−0.16 (0.21)	−0.07 (0.14)	−0.22 (0.19)
		*R*^2^ = 0.10		*R*^2^ = 0.03		*R*^2^ = 0.02				
		*F*(2, 57) = 3.79 *		*F*(2, 57) = 0.66		*F*(2, 57) = 0.55				
M (ERS)										
M1 (CS)		–	–	–	–	–	*b*_1_	0.28 (0.31)	0.01 (0.20)	0.16 (0.30)
M2 (PB)		–	–	–	–	–	*b*_2_	−0.26 (0.23)	−0.19 (0.15)	−0.21 (0.20)
M3 (PMT)		–	–	–	–	–	*b*_3_	0.05 (0.24)	0.21 (0.13)	0.18 (0.20)
W (GRS Beliefs)		–	–	–	–	–	*b*_4_	0.25 (0.41)	−0.06 (0.27)	−0.11 (0.36)
X × W		–	–	–	–	–	*b*_5_	0.23 (0.69)	0.09 (0.43)	0.30 (0.68)
M1 × W		–	–	–	–	–	*b*_6_	−0.40 (0.34)	−0.03 (0.24)	0.05 (0.39)
M2 × W		–	–	–	–	–	*b*_7_	0.54 (0.28) ^†^	0.37 (0.21)	0.22 (0.33)
M3 × W		–	–	–	–	–	*b*_8_	−0.47 (10.65)	−0.45 (10.01)	−0.39 (10.71)
								*R*^2^ = 0.24	*R*^2^ = 0.24	*R*^2^ = 0.24
								*F*(10, 49) = 2.17 **	*F*(10, 49) = 2.17 *	*F*(10, 49) = 2.23 *
						**Moderated mediation**		Index, 95% CI	Index, 95% CI	Index, 95% CI
						M1 (CS)		−0.02 [−0.18, 0.09]	0.00 [−0.10, 0.07]	−0.00 [−0.07, 0.07]
						M2 (PB)		0.02 [−0.12, 0.22]	0.01 [−0.08, 0.11]	0.01 [−0.08, 0.14]
						M3 (PMT)		−0.05 [−0.24, 0.08]	−0.04 [−0.21, 0.05]	−0.04 [−0.20, 0.07]

*Note*. All models were bootstrapped (10,000 samples). N = 60. * *p* < 0.05 ** *p* < 0.01 *** *p* < 0.001 ^†^ *p* = 0.05 (marginal).

**Table A2:** Regression coefficients for neighborhood problems models 4–6.

Neighborhood Problems Models (4–6)							
	Consequent						
	(a-path is the same for models 4–6)				**Model 4**	**Model 5**	**Model 6**
	*M*_1_ (CS)	*M*_2_ (PB)	*M*_3_ (PMT)		*Y*_1_ (Centrality)	*Y*_2_ (Private Regard)	*Y*_3_ (Public Regard)
Antecedent	Coeff. (*SE*)	Coeff. (*SE*)	Coeff. (*SE*)		Coeff. (*SE*)	Coeff. (*SE*)	Coeff. (*SE*)
Constant	−0.56 (0.25) *	−0.27 (0.27)	0.18 (0.27)		0.28 (0.31)	0.11 (0.24)	0.42 (0.22)
X (RN Problems)	0.00 (0.17)	0.28 (0.15)	0.18 (0.20)	*c*’	−0.25 (0.23)	−0.03 (0.14)	−0.07 (0.15)
Z (Parent Education)	0.28 (0.10) **	0.14 (0.11)	−0.09 (0.11)		−0.15 (0.12)	−0.06 (0.09)	−0.21 (0.09) *
	*R*^2^ = 0.10	*R*^2^ = 0.09	*R*^2^ = 0.04				
	*F*(2, 57) = 3.65 *	*F*(2, 57) = 2.51	*F*(2, 57) = 0.76				
M (ERS)							
M1 (CS)	–	–	–	*b*_1_	0.29 (0.19)	0.04 (0.14)	0.19 (0.17)
M2 (PB)	–	–	–	*b*_2_	−0.25 (0.19)	−0.22 (0.13)	−0.23 (0.16)
M3 (PMT)	–	–	–	*b*_3_	0.12 (0.21)	0.25 (0.13) ^†^	0.25 (0.16)
W (GRS Beliefs)	–	–	–	*b*_4_	0.26 (0.24)	−0.01 (0.16)	−0.05 (0.17)
X × W	–	–	–	*b*_5_	−0.61 (0.38)	−0.56 (0.24) *	−0.57 (0.27) *
M1 × W	–	–	–	*b*_6_	−0.35 (0.33)	−0.14 (0.23)	0.04 (0.36)
M2 × W	–	–	–	*b*_7_	0.58 (0.29) ^†^	0.40 (0.21) ^†^	0.27 (0.34)
M3 × W	–	–	–	*b*_8_	−0.12 (0.38)	−0.09 (0.31)	−0.07 (0.25)
					*R*^2^ = 0.30	*R*^2^ = 0.26	*R*^2^ = 0.28
					*F*(10, 49) = 3.12 **	*F*(10, 49) = 1.61	*F*(10, 49) = 1.82
			**Moderated mediation**		Index, 95% CI	Index, 95% CI	Index, 95% CI
			M1 (CS)		0.00 [−0.17, 0.19]	0.00 [−10, 0.10]	0.00 [−0.10, 0.11]
			M2 (PB)		0.16 [−0.03, 0.54]	0.11 [−0.03, 0.34]	0.08 [−0.02, 0.37]
			M3 (PMT)		−0.02 [−0.29, 0.15]	−0.02 [−0.21, 0.12]	−0.01 [−0.19, 0.09]

*Note*. Percentile bootstrap CI based on 10,000 bootstrap samples. N = 60. ^†^ *p* < 0.06 (marginal); * *p* < 0.05. ** *p* < 0.01.
